# Biologically inspired load balancing mechanism in neocortical competitive learning

**DOI:** 10.3389/fncir.2014.00018

**Published:** 2014-03-11

**Authors:** Amir Tal, Noam Peled, Hava T. Siegelmann

**Affiliations:** ^1^The Leslie and Susan Gonda Multidisciplinary Brain Research Center, Bar-Ilan UniversityRamat-Gan, Israel; ^2^The Biologically Inspired Neural and Dynamical Systems Laboratory, Computer Science Department, University of MassachusettsAmherst, MA, USA

**Keywords:** competitive learning, layer 5 pyramidal cells, STDP learning, brain circuitry development, dead units problem

## Abstract

A unique delayed self-inhibitory pathway mediated by layer 5 Martinotti Cells was studied in a biologically inspired neural network simulation. Inclusion of this pathway along with layer 5 basket cell lateral inhibition caused balanced competitive learning, which led to the formation of neuronal clusters as were indeed reported in the same region. Martinotti pathway proves to act as a learning “conscience,” causing overly successful regions in the network to restrict themselves and let others fire. It thus spreads connectivity more evenly throughout the net and solves the “dead unit” problem of clustering algorithms in a local and biologically plausible manner.

## Introduction

The neocortex is probably the most magnificent piece of biological machinery we possess, responsible above all else for making us who we are both as a species and as individuals. Structural studies reveal it is made of a relatively repeating stereotypical hierarchy of columns and layers, slightly varying between different regions in charge of different tasks. Closer inspection, however, hits an “impenetrable jungle” of connectivity, leaving neocortical inner-circuitry largely a mystery to this day.

Recently, several small-scale neocortical connectivity structures have been found, one of which is a unique inhibitory pathway of sub-columnar dimensions. It provides prolonged delayed feedback, inhibiting excitatory pyramidal cell (PC) neighborhoods which stir it to action within a distinctive delay from activity onset (Kapfer et al., 2007; Silberberg and Markram, 2007; Berger et al., 2010). This pathway is mediated by Martinotti cells (MC), a somatostatin (SOM)-expressing prominent member of the neocortical interneuron population, most frequent in layer 5 (Markram et al., 2004). In the same 5th layer, other studies have found recurring structures and regularities in PC network connectivity (Song et al., 2005; Perin et al., 2011). Across different animals, similar patterns of PC clustering were found in a remarkably predictable fashion. Finding such regularities, in postnatal animals especially, begged the question of their origin—might they be predetermined, perhaps DNA prescribed?

In describing layer 5 circuitry, a third important player is missing. Large basket cells (LBC) are the most prevalent interneuron type of the entire neocortex and of layer 5 specifically. They are parvalbumin (PV) expressing, and together with MC, these two types constitute the vast majority of the layer's interneurons (Markram et al., 2004; Rudy et al., 2011). In contrast to MC's local innervation, LBCs are known to be the most common lateral inhibition neurons of the neocortex (Wang et al., 2002; Markram et al., 2004), extending expansive lateral axonal arborizations to neighboring and distant columns within their layer. For this reason, they are frequently attributed imposing Mexican hat shaped inhibition in the neocortex (Casanova et al., 2003), dynamics which are prominent in neural network literature (Kang et al., 2003) and constitute a basic circuitry principle in the cortex, mapping cortical input to output (Adesnik and Scanziani, 2010).

Putting together PC neurons with MC and LBC inhibition, a comprehensive simulation of neocortical layer 5 circuitry is possible, covering approximately 95% of the layer's neuronal types. We have composed such a neural network simulation and examined how these novel inhibition dynamics affect network behavior and development. We have found that the combination of MC and LBC inhibition produces a unique balance in learning. MC inhibition with its spatial and temporal traits constitutes a substantial addition to classic local-excitation lateral-inhibition connectivity, acting as a chief regulator of competitive learning. A useful competitive circuit is formed, which under simple spike-timing dependent plasticity (STDP) rules inevitably develops clustered connectivity patterns in an excitatory PC network, patterns which were indeed found to exist.

Although much has been discovered in recent years on both the importance and the characteristics of cortical inhibitory neurons, understanding of their circuitry and subtype-specific role in cortical computation is so far lacking (Isaacson and Scanziani, 2011). In this study we therefore offer a feasible interpretation of some of the function both LBCs and MCs play in cortical dynamics, both in agreement with previous work ascribing them with input selectivity and tuning responsibilities.

## Materials and methods

### Single neuron simulation

Leaky integrate-and-fire (LIF) model (Burkitt, 2006) was chosen to simulate PC neurons in this study due to its biological relevance along with computational simplicity, according to the following update equation:

(1) $$V(t+\Delta t)=\left(1-\frac{\Delta t}{\tau }\right)V(t)+\frac{\Delta t}{C}I(t)+\frac{\Delta t}{\tau }V_{\text{rest}}$$

Membrane resting potential *V*_rest_ is set to −65 mV, threshold potential to −40 mV and time constant τ to 20 ms, based on the electrophysiological characteristics of cortical pyramidal neurons. Firing is followed by an absolute refractory period of 1 ms.

### Network input

At any given time of the simulation, a randomly generated input is driving the network. A single input is in fact a binary vector of size N, so all neurons act as channels to the outside world to this extent. An input pattern is held constant for a time window of 160 ms so encompassing 4 learning iterations (see Plasticity), after which it is regenerated, switching on/off each of the N input channels at an independent probability of 0.5 each. An active input channel mimics an electric current producing a driving force of 5 mV EPSP, which causes stimuli receiving neurons to fire at a frequency of 166 Hz. Stemming from Equation 1, in order to achieve a PSP change of γ (in mV), external input at time *t* should be:

(2) $$I(t)=\left\{\gamma +\frac{\Delta t}{\tau }V(t)-\frac{\Delta t}{\tau }V_{\text{rest}}\right\}\frac{C}{\Delta t}$$

Assuming post-synaptic neuron is at resting potential at time *t*, γ PSP would be achieved within Δ*t* time by an outside current of:

(3) $$I(t)=\gamma \frac{C}{\Delta t}$$

Synaptic weights are calculated using Equation 3, with Δ*t* of 1 ms in order to produce the effect by a single pre-synaptic spike at resting potential.

### Simulation procedure

A standard simulation begins with random distance-dependent connectivity between neurons. Timeline is dependent primarily upon learning rate, a variable dictating the extent to which learning affects the network. Learning rate declines exponentially over time, reflecting network stabilization. This enables the network to change dramatically on early stages of simulation and then slowly stabilize and fine tune, in a way resembling AI self-organizing maps (SOMs). Simulation ends when learning becomes practically ineffective. Standard learning rate of our simulation declines at a factor of 550. Update is done in learning iterations lasting 40 ms each, thus allowing 885 such iterations throughout a single simulation.

### PC circuitry

Simulation network is made of N pyramidal neurons (1000 as standard), with connectivity matrices representing excitatory and inhibitory connections. Only PCs are implemented as model spiking neurons so to keep the simulation relatively simple, and portray the key effect on PC interconnectivity arising from MC-LBC structure dynamics (see Results, for a discussion of this approach). Networks represent a 3D cube of cortical tissue, so each PC is ascribed a three dimensional coordinate. Average distance between neurons is set to 36 μm between cortical PCs (based on unpublished electrophysiological findings), while an additional position jitter of up to 30 μm is randomly applied. Given the network has within it a maximal distance of *maxDist*, connectivity is initialized naively distance dependent using the following connection probability for any two neurons *I* and *j*:

(4) $$P\left({\text{connect}}_{i,j}\right)={\left(\frac{maxDist-{\text{dist}}_{i,j}}{maxDist}\right)}^{5}$$

Because distances distribute normally around 250 μm, Equation 4 results in 12.2% connectivity initialization on average. PC-PC synapses undergo synaptic efficacy molding throughout the simulation, and are randomly initialized between 1 and 4 mV EPSP.

### LBC circuitry

Basket Cell inhibition in the net is expressed by depressing connections made between PCs. LBC-PC synaptic strength in our study is set to 1.5 mV IPSP (Thomson et al., 2002). Inhibitory BC connectivity between PCs is set, again, following distance-dependent connection probability, normally distributed around a mean distance of 40 μm with a variance of a third of the maximal distance (*maxDist/*3). This is done to match the geographical distribution and connection probability of somatosensory layer 5 LBCs taken from Packer and Yuste (2011).

### MC circuitry

Delayed self-inhibition, the inhibitory pathway attributed to MCs is manifested in the model as a paralyzing inhibitory current affecting a 50 μm neighborhood range of an active PC neuron (Silberberg and Markram, 2007). Each PC is a center of such a neighborhood, initiating a 50 μm radius inhibition around it when active, so to not bias to begin with formation of any specific neighborhoods. MC inhibition lasts 120 ms but within 240 ms delay from activity onset (Silberberg and Markram, 2007).

### Plasticity

Synaptic plasticity in the simulation comes in two major forms, both governed primarily by the global learning rate. The primary form of plasticity is the ongoing learning process of STDP, modulating synaptic strength according to its directionality and according to the relative timing of spikes coming from the two neurons on both ends of it (Feldman, 2012). For this research a classic “Hebbian” STDP function was implemented from Bi and Poo (1998) with two major modifications:

1. Effective time slot for LTD and LTP are unsymmetrical, depression sensitive to larger time scales than potentiation, as several studies have found (Feldman, 2000; Sjöström et al., 2001).
2. Certain randomness is incorporated for determining exact volume of synaptic change. As can also be seen in Bi and Poo (1998), classic STDP function defines a range, not a deterministic value of change per each Δ*t*. This is probably due to a multitude of factors which influence actual STDP in addition to pairwise directionality and timing, such as firing rate, synaptic location, and trains of multiple spikes (Pfister and Gerstner, 2006; Froemke et al., 2010a,b; Feldman, 2012)

Using an LTP window φ_1_ of 20 ms along with an LTD window φ_2_ of 40 ms, the following equation describes the change applied to synapse *A*_*i,t*_ as the result of a time difference of Δ*t* ms between a presynaptic spike from neuron *I* and a postsynaptic spike from neuron *t* (where *r* is a random scalar between 0 and 1 embodying randomness described above in point 2, and δ is the current global learning rate):

(5) $$A_{i,t}=\{\begin{array}{l} A_{i,t}\;+\;0.05\;*\;A_{i,t}\;*\;e^{\left(\frac{-△t}{30}\right)}\;*\;r\;*\;\delta \;0\leq △t<\varphi_{1}\\ A_{i,t}\;-\;0.05\;*\;A_{i,t}\;*\;\frac{e^{\left(\frac{△t}{30}\right)}}{2}\;*\;r\;*\;\delta \;-\varphi_{2}<△t<0 \end{array}$$

A 0.05 factor is applied to make single spike change very small and in fact resemble real plasticity in which a train of at least 20 spikes is required for functional change.

A second separate form of plasticity affects inactive synapses rather than active ones in a periodic fashion. A learning iteration window is defined (40 ms), at the end of which synapses which have not been altered by STDP in the last period undergo a “natural” small weakening.

Synapses in the simulation are allowed to vary in strength between 0.05 and 12 mV EPSP. Upper bound constitutes synapse saturation and cannot be exceeded. Lower bound determines threshold for pruning away the synapse.

### Analysis

Result analysis is done using custom Matlab software and python scripts. For cluster connectivity comparison and analysis we use Affinity Propagation (AP) algorithm in this study (Frey and Dueck, 2007). AP was chosen in order to submit simulation results to the same tests used by Perin et al. (2011), and so provide the best possible comparison between the two. AP algorithm finds the best matching representatives (termed “exemplars”) of a set of data points, given their similarity to one another and a value for each point indicating the preference of it being chosen as such an exemplar. A subset of exemplars is eventually converged upon, optimizing input values. Exemplars together with the neurons they represent constitute clusters. For our study, similarity between each pair of neurons is defined as the number of common neighbors (NCN) shared by them, and preference value is set to a common value of 2, as was done by Perin et al.

For firing pattern analysis, we used Manhattan distance to assess spike train similarity. This metric provided results similar to those of the Victor-Purpura spike train metric (Dauwels et al., 2009)^1^, which is infeasible to calculate for large networks within reasonable time. PCA algorithm was used on spike train distance matrix in order to reduce feature space dimensionality, and the first five components of PCA were picked, explaining 99% of distance variance. Over this N × 5 features space we applied mixture of Gaussians clustering method (Titterington et al., 1985) to find neurons with similar spiking behavior. Only the last quarter of simulation activity was regarded in order to analyze evolved network dynamics.

To determine the most reasonable number of clusters emerging from our data, we used information-theoretic criteria (BIC) (Burnham and Anderson, 2002) and chose the model bearing minimal BIC score. Two distinct types of clusters were found—clusters of correlated active neurons and clusters of silent ones, which almost did not fire and were therefore disregarded from analysis. For oscillation analysis, spike trains of the most active unit of each cluster were examined. Auto-correlation of these spike trains revealed oscillatory behavior, while cross-correlation between all pairs revealed phase differences and alternation patterns.

## Results

**Figure 1:**
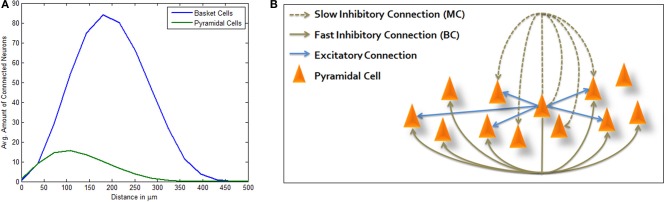
**Three-way inhibitive structure. (A)** Average amount of connected PCs (green) and LBCs (blue) from a single PC per inter-somatic distance (avg. sum of neurons per distance surrounding a single PC). PC and LBC distributions and connectivity functions allow for Mexican hat shaped excitation, as connected PCs are generally closer than connected LBCs. **(B)** Illustration of structure topology. Activation of central PC immediately drives connected PCs while inhibiting others which are connected via basket cell lateral inhibition. It also inhibits its own local neighborhood within a certain delay, the effect attributed to Martinotti cells.

Following empirical geographical distributions and connection probabilities (see Materials and Methods), connected LBCs are more distant from the average PC than connected PCs are from it (Figure 1A) allowing Mexican hat shaped excitation. Adding MC inhibition produces the complete structure depicted in Figure 1B. As described in Materials and Methods, simulation is highly simplified, using LIF model for PC neurons and modeling inhibitory neurons only as inhibitory currents lacking neuronal dynamics of their own. Many network connectivity complexities are disregarded in this way, such as synapse targeting location and inhibition of inhibition, however it allows focused portrayal of a computational principle and its abstraction from finer scaled anatomical debate. We chose this approach in order to bridge between biological and computational knowledge, hoping each could afford some insight to the other. Our model was run under random input and through different sized simulations. End-point network connectivity was analyzed and compared to its distance-dependent random initialization.

### Clustering effect

**Figure 2:**
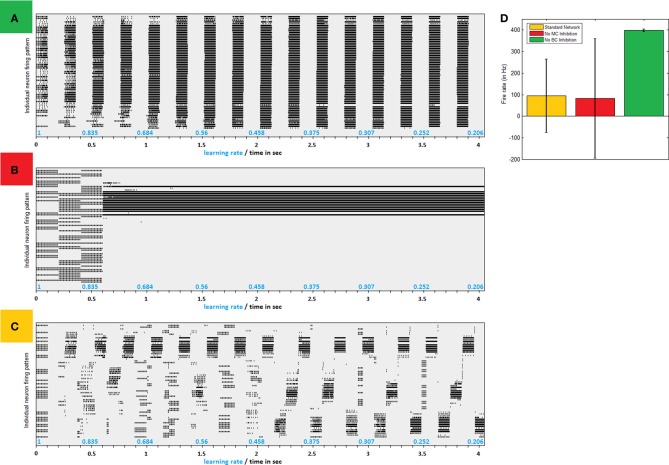
**Inhibitive structure effects—a comparison of firing patters produced by same network without LBC inhibition, without MC inhibition and with both inhibitions in place. (A)** Firing raster plot of a 64 neuron network absent LBC inhibition. In early stages firing is according to input pattern. Very quickly, more and more neighboring neurons are recruited until all fire regardless of input. Only breaks in activity are offered by MC inhibition, which becomes in sync between all neurons. **(B)** Firing raster plot of a network absent MC inhibition. Network dynamics eventually lock down to one area, allowing it to grow perpetually stronger and remain active, while shutting down others with no real competition. **(C)** Firing raster plot of similar network with full structure in place. Firing gradually converges to separate regions of clustered synchronized activity **(D)** Avg. firing rate in different paradigms. Lack of MC (red bar) leaves firing rate similar to normal network (in yellow) but at a greater variance as network becomes uneven. Removal of LBC inhibition, on the other hand, results in homogeneous huge volumes of firing (green).

Connectivity as illustrated in Figure 1B causes PC network to gradually converge to separate synchronized competing regions, as can easily be seen in firing patterns produced by simplified models of small networks (Figure 2C). Clustered activity is not surprising under “Mexican hat” shaped inhibition as imposed by LBC inhibition. Indeed, networks without this type of cell exhibit a steady influx of activity until almost saturation (Figure 2A). However, clustering balance is apparently lost when MC inhibition is removed from dynamics.

### Role of MC inhibition

**Figure 3:**
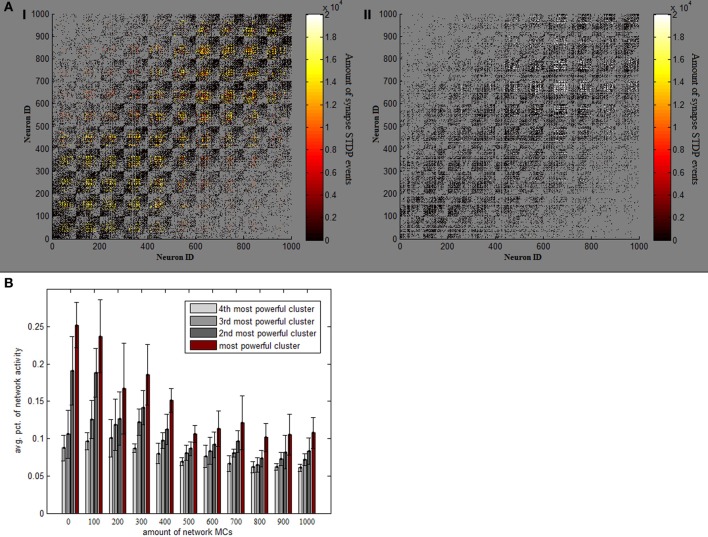
**Effect of MC inhibition. (A)** Temperature plots indicate the amount of STDP events which each synapse underwent in one full 1000 neuron simulation, on network connectivity matrices. Panel **I** (on the left) depicts a standard simulation, while panel **II** is an example of a simulation lacking MC inhibitive pathway. Removing MC ruins the even spread of STDP events and causes them all to happen in one rather confined area (in very high volumes). **(B)** Avg. percentage of overall network activity is plotted for each of the 4 most active clusters, per different amounts of MCs in the network. As more and more MCs are added, activity becomes more balanced between clusters.

Absent MC inhibition, one area of the network will eventually reach a critical mass of strength, which will cause it to be the most driven region in the net for any outside stimulus. This region breaks through dynamics and grows unequivocally dominant with less and less competition (Figure 2B). The product of such a network is therefore heavily unbalanced, as an average 5% of neurons produces around a fourth of network activity and inequality between firing rates is thus substantially larger (Figure 2D). This dominant group's geographical locality is evident in analyzing plasticity events the network underwent (Figure 3A). STDP activity does not spread equally across the net but is rather restricted to a confined selected area, which produces all the firing activity. Varying the amount of MCs in the network (randomly selecting a subset of PCs activating MC inhibition) reveals the graded effect this inhibition has on network dominance (Figure 3B). The more MC inhibition in the network the more balanced it becomes, reducing activity disparity between clusters and their overall domination of the net.

It appears MC inhibition has a regulatory role in competitive dynamics. It enforces certain synchronization in inhibition of pyramidal neighborhoods (Berger et al., 2010). When a region becomes active and strengthens it is MC inhibition which is in charge of shutting it down, allowing other areas to grow strong as well. Both LBC and MC inhibition play a vital role in cluster formation, therefore. It is LBC structure which imbeds an inherent tendency to cluster, and MC regulation of it which makes that tendency well-spread and useful throughout the entire network. If LBC inhibition causes groups of neurons to compete with one another, MC inhibition makes that competition fair.

### Comparison with neocortical clusters

Using the same paradigm used in real brain tissue analysis (Perin et al., 2011), we next compared the clustering effect of LBC-MC inhibition to empirically found connectivity traits, in order to examine the possible relation between the two.

Full length simulation results in the elimination of a little under half of all synapses, somewhat resembling cortical development between early childhood and puberty (Chechik et al., 1998). Connection probability remains distance dependent, but, in general, connections become shorter and stronger, more “focused” so to speak. AP analysis (see Materials and Methods) yields 40.4 ± 1.71 clusters at an average size of 24.8 ± 1.06 members each. For comparison, a rerun of the simulation by Perin et al. (2011) for 1000 neurons results in clusters of 31.58 ± 2.29 members.

**Figure 4:**
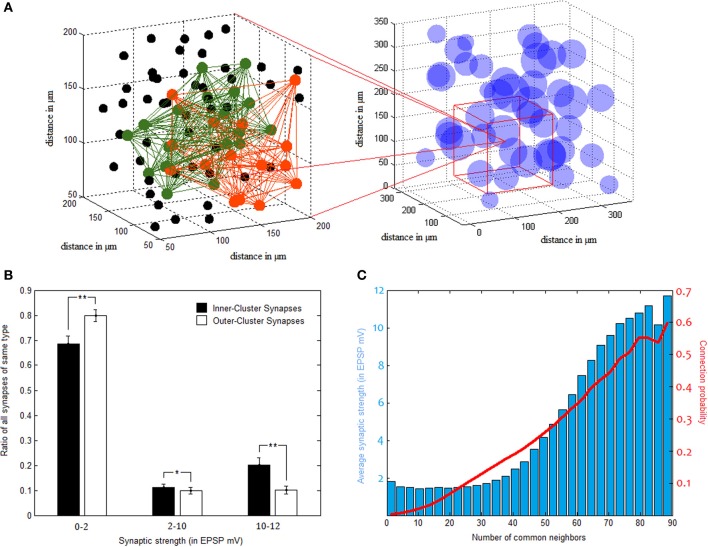
**Pyramidal clusters. (A)** Illustration of cluster overlap. On the right—cluster distribution in 3d space. Clusters are drawn as spheres using their calculated center point and average diameter. They overlap substantially, as can be seen zooming in to a region (on the left hand side). Two different clusters, colored green and orange, interlace in this space. Black dots indicate neurons which belong to neither of the two clusters. **(B)** Strong synapses constitute a larger fraction of inner-cluster synapses than outer-cluster synapses (STD bars shown in black. ^*^p < 0.05, ^**^p < 0.001). **(C)** Common neighbor rule. Both the probability to connect (in red) and the average connectivity strength (blue bars) rise as a function of the number of neighbors two PC neurons share.

Clusters in our network comprise an average connectivity ratio of 30.06 ± 0.86%, almost four times higher than the overall network ratio. These densely connected clusters, however, overlap substantially in between themselves as well, allowing vast cooperation possibilities between one another (as illustrated in Figure 4A). In terms of synaptic strength, while there are far more synapses bridging different clusters than those connecting neurons of the same cluster, synaptic strength tends to distribute rather similarly in the two groups, with tremendous variance in both. This bimodal distribution of synaptic weights is common in naïve forms of STDP learning, and may be balanced using additional parameters to plasticity (Van Rossum et al., 2000), a complexity we thought unnecessary for our model. Despite this large variability, strong synapses constitute a significantly higher percentage of inner-synapses than outer-synapses (Figure 4B). Synaptic strength inside clusters is on average 3.43 ± 0.31 mV (in EPSP), while the average synapse outside clusters is of 2.29 ± 0.18 mV EPSP strength.

NCN was found by Perin et al. (2011) to constitute a stable organizing principle for network connectivity, reliably predicting both connection probability and strength between pairs of pyramidal neurons. It was used in this study for cluster identification in the same manner that was done in the original paper. In light of similar tendencies found and reported above, NCN rule proves to apply to our networks as well. This is evident in Figure 4C, as the more common neighbors two neurons share the more likely they are to be connected, and the stronger the connection between them tends to be.

### Firing patterns

**Figure 5:**
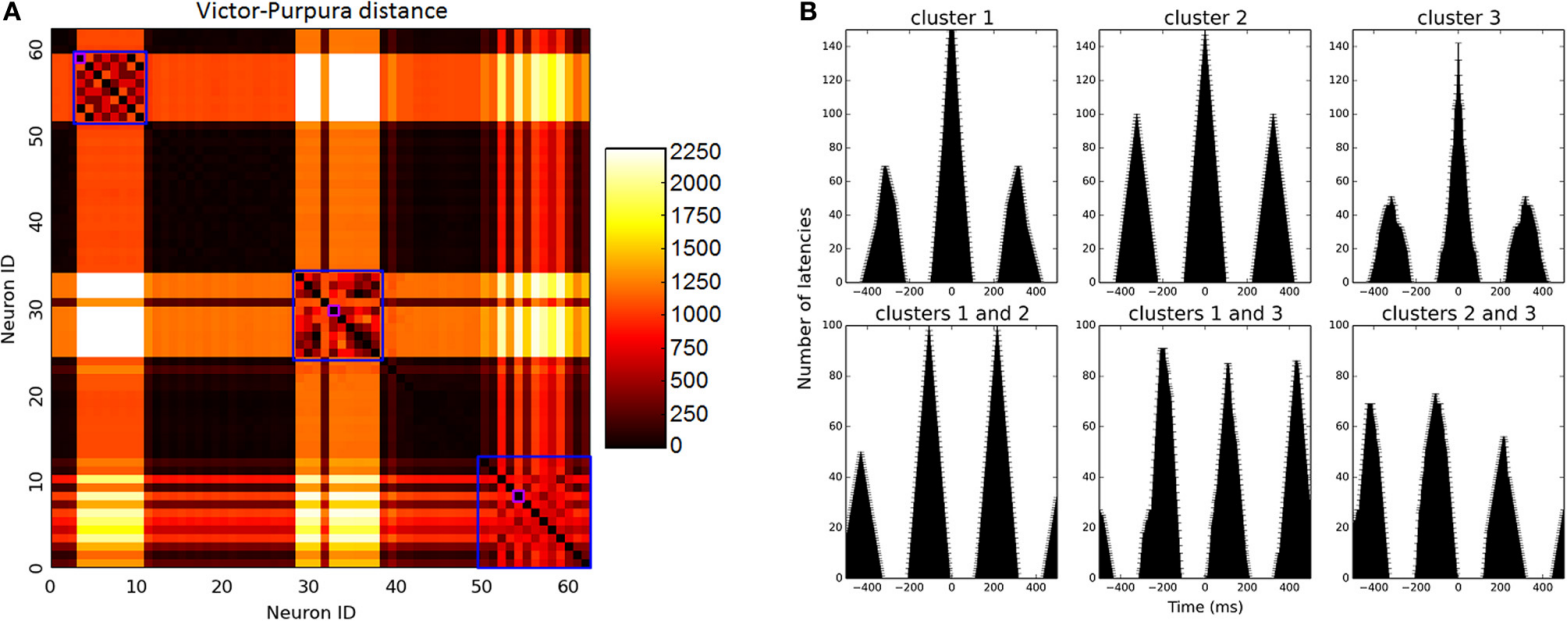
**Firing pattern analysis. (A)** Matrix of Victor-Purpura distance between each neuron to all others. Clusters were identified using MOG algorithm, marked on the figure in blue squares. Of each cluster, the most active unit was selected to represent firing rate, marked in purple for illustration. **(B)** Firing analysis of a sample 64 neuron network. Upper row depicts auto-correlation of neurons from 3 prominent clusters (i.e., amount of intervals that occurred between spikes per different time windows). Oscillatory behavior is manifested in repeating auto-correlation interval peaks (around 300 ms). On the bottom row, cross-correlation between neurons of different clusters illustrates alternating activity between clusters. Activity oscillates between clusters at different intervals.

Since biologically resembling connectivity tendencies emerge, it is interesting to examine network firing patterns, as such mini-scale behaviors are so far inaccessible in real live brain tissues. Network activity reaches high gamma frequency firing rates (90 Hz on average) and becomes rather oscillatory. Prominent clusters which have emerged during development compete for input and alternate in activity in between them, firing rather synchronously. In small networks, 2–3 clusters emerge as dominant and share the bulk of network activity in between them (Figures 2C, 5A). In large networks lateral inhibition extends to a smaller portion of the network and dynamics are more complex, creating 4–5 larger prominent clusters which overlap in activity. These clusters span the entire network but with an average inter-somatic distance favoring neighbors (112.4 ± 4.8 μm).

Cluster oscillatory behavior is evident in triangular-like auto-correlation analysis of their spiking activity (Figure 5B, upper row), and alternation between clusters evident in cluster cross-correlation (Figure 5B, bottom row). Oscillatory time scale is highly dependent upon MC temporal dynamics and the joint activation time window they allow. These temporal dynamics are over-simplified in our model and oscillations would be expected to be much more varied in real dynamics, however recent findings have shown that MCs do in fact behave in an oscillatory fashion themselves as they do in our model, enhancing excitability and alternating layer 5 PC firing mode (Li et al., 2013).

## Discussion

In this study we have shown that a unique inhibitory feedback structure found in the neocortex may contribute in forming a useful network circuit, embedding well-regulated competitive learning within the net. Generalization of this architecture to the entire cortex is questionable, as we know different areas harbor specialized circuits. However, lateral inhibition as described in this paper and the general structure of Martinotti inhibition are widely prevalent throughout the cortex (Kätzel et al., 2011), and so the dynamics described in this paper may be a general outline of circuit behavior, subject to local variations. The inhibitory pathway of MC has been reported both between layer 2/3 and layer 5 PCs (Adesnik and Scanziani, 2010), and in between layer 5 PCs themselves (Silberberg and Markram, 2007). Both of the above would contribute to layer 5 PC tuning and would so predict the formation of regional layer 5 PC clusters, as were indeed found to exist.

### Martinotti as learning conscience

Self-inhibition in the form described by Silberberg et al. (Silberberg and Markram, 2007) and naively incorporated in this study has proven to be an important addition to local-excitation lateral-inhibition dynamics. Delayed self-inhibition acts as a competition regulator, inflicting restraint to powerful areas of activity and so allowing other regions to compete fairly in between them for response to following inputs. This regulation results in the formation of many evenly distributed clusters instead of few dominant ones, solving, in fact, the “dead unit” problem prevalent in competitive learning paradigms.

“Dead units” are output units which do not eventually contribute to computation, units which did not win enough competitions and end up not representing anything in the input space (usually due to bad initializations) (Xu et al., 1993). This affliction is frequently suffered by hard competitive learning algorithms, amongst others by classic SOM which our network somewhat resembles in learning behavior. Extensions to SOM and other techniques were developed in the field of AI to overcome this shortcoming (Van Hulle, 2012).

Several approaches, most notably “neural gas” and its offshoots (Martinetz and Schulten, 1991; Fritzke, 1995), addressed the dead unit problem by relaxing predefined constraints. Adding or removing neurons online or rearranging locations freely allowed them to converge onto an ideally utilized topology based on cooperative interactions between neurons. Other approaches left topology fixed and instead addressed the nature of competition itself by, for instance, considering neurons' previous number of wins (Ahalt et al., 1990), penalizing 2nd place winners (Xu et al., 1993) or imbedding a “bad conscience” to frequent winners which brings on self-restriction (DeSieno, 1988). This last method is very reminiscent of MC behavior observed in this study. It bears the same effect on learning and the same rational on a system point of view, but most importantly it achieves global regulation using only simple local conditions. Due to MC delayed temporal dynamics, this inhibitive pathway manages to restrict excessively successful regions while being ignorant of the grand network scheme and learning history, and without requiring radical topological rearrangement. Both latter AI approaches would be implausible if not impossible in a living biological system.

Following the analogy of competitive learning, the necessity for many well-spread clusters over few dominant ones is clear. Competitive learning allows regions to grow separately suited for different inputs. “Winning” response to an input means growing more attuned to that certain stimulation and so, on the long run, ideally matching it. Such input specification is lost if one region “wins” every consecutive input. This one region would eventually be strong enough to match any given input, and therefore no separation of inputs will be achieved by the network, and no learning at all.

### Interlaced clusters

PC clusters were postulated to constitute elementary building blocks of cognition. Individual experience, by this account, mixes and matches such clusters to endless possible combinations, but clusters themselves will always underlie such a structure, embodying a theoretical limit to the freedom Hebbian plasticity has over the network (Perin et al., 2011). This view is supported by our findings, as it suggests pyramidal clusters are an outcome of activity rivalry and input selectivity. Internal wiring causes clusters to develop, competing with one another for varying patterns in external input. Granted an ever-existent plastic ability (embodied in our model as a learning rate which diminishes but is never zero) experience will mold clusters throughout life. This will form different clusters in different animals in different times, always subject to dynamic change and rewiring to some extent, but a clustered formation is in anyway inevitable.

As was also reported in Perin et al. (2011) clusters appear to be highly involved in one another. Certain groups of neurons connect preferentially over time with many highly influential connections between one another. However, they span a relatively large space in the cortex, interlacing with other groups of neurons and connecting to them as well. Therefore, clusters are by no means separate islands of activity which simply transmit a binary all-or-nothing output between them, but are rather cooperative units, which have vast combinatorial possibilities to connect and affect one another.

### Connection specificity

The developmental process described in this paper allows for network connectivity to be formed by chance. Although dependent only upon distance, connectivity in the simulation inevitably creates larger more complex and repeating structures which would otherwise seem non-random. This is all due to cell morphology traits, i.e.: MC long ascending axon and LBC extensive axonal branching, and no higher more complex design. It therefore constitutes something of a compromise in the debate of neuronal connectivity specificity (Hill et al., 2012).

Due to the intricacy of cortical wiring, it is still largely unknown to what degree is neuronal connectivity specific (Fino and Yuste, 2011). Some studies have found repeating circuits and connectivity tendencies, supporting a view of highly predetermined connectivity (Thomson and Lamy, 2007), while others report a more promiscuous form of connectivity forming a “tabula rasa” network for experience to mold (Fino and Yuste, 2011; Packer and Yuste, 2011). Under our paradigm, although circuits are rather mature, repeating and stable, and layer 5 pyramidal network conforms to an organizing principle of predictable traits, no innate goal-oriented connectivity is needed. Instead, these patterns are a natural outcome of simple Hebbian plasticity, under the influence of different types of inhibitory cells. These cells' basic morphology and location will amount to useful complex structures.

### Conflict of interest statement

The authors declare that the research was conducted in the absence of any commercial or financial relationships that could be construed as a potential conflict of interest.

## Footnotes

^1^Victor-Purpura distance metric defines the distance between two spike trains as the minimum cost of transforming one train to the other using three basic operations: insertion, deletion, and movement. Using this metric, each neuron is defined as an N vector space, where the i'th element is its Victor-Purpura distance from the i'th neuron.
